# Lokale Lappenplastiken als letzter Versuch vor der Unterschenkelamputation: eine Übersicht

**DOI:** 10.1007/s00113-020-00814-6

**Published:** 2020-05-08

**Authors:** Rafael G. Jakubietz, Rainer H. Meffert, Michael G. Jakubietz, Florian Seyfried, Karsten Schmidt

**Affiliations:** 1 Klinik für Unfall‑, Hand‑, Plastische und Wiederherstellungschirurgie, Universitätsklinikum Würzburg, Oberdürrbacherstr. 6, 97080 Würzburg, Deutschland; 2 Klinik und Poliklinik für Allgemein‑, Viszeral‑, Transplantations‑, Gefäß- und Kinderchirurgie, Universitätsklinikum Würzburg, Würzburg, Deutschland

**Keywords:** Gefäßgestielte Lappenplastik, Multimorbide Patienten, Suralislappenplastik, Peronaeus-brevis-Muskellappenplastik, Perforatorlappenplastik, Pedicled flap reconstruction, Multimorbid patients, Sural artery flap, Peroneus brevis muscle flap, Perforator flap

## Abstract

**Hintergrund:**

Die Weichgewebsrekonstruktion bei alten Patienten stellt eine Herausforderung dar. Der freie Gewebetransfer kann bei gesunden Patienten trotz hohen Alters mit hoher Erfolgsrate durchgeführt werden. Bei multimorbiden Patienten, die für den freien Gewebetransfer nicht in Betracht kommen, werden häufig lokale Lappenplastiken eingesetzt, welche mit einer hohen Komplikationsrate assoziiert sind. Gerade solche Rettungseingriffe müssen so gewählt werden, dass eine Amputation durch die Wahl der Entnahmestelle nicht nachteilig beeinflusst wird oder gar unmöglich wird.

**Methode:**

Drei distal basierte lokale Lappenplastiken wie Suralis‑, Peronaeus-brevis- und Propellerlappenplastik werden im Hinblick auf die Platzierung der Entnahmestelle sowie die Komplikationen der Wundheilungsstörung diskutiert.

**Ergebnisse:**

Die Entnahmestelle der Suralislappenplastik ist nachteilig, da die proximale, dorsale Wadenregion betroffen ist, die im Falle einer Unterschenkelamputation die Weichteildeckung des Stumpfes ermöglicht.

**Schlussfolgerung:**

Soll eine lokale Lappenplastik als „Rettungseingriff“ als Versuch der Vermeidung einer Unterschenkelamputation bei für einen freien Gewebetransfer nichtgeeigneten Patienten zum Einsatz kommen, gilt es, Lappenplastiken zu wählen, die die Entnahmestelle außerhalb der dorsalen, proximalen Wade platzieren, um die Weichteildeckung einer Stumpfbildung potenziell zu ermöglichen.

## Abstract (en)

**Background:**

Soft tissue reconstruction in aged patients is challenging. Free tissue transfer can be carried out in healthy patients with a high success rate despite old age. When free tissue transfer is contraindicated in multimorbid patients, local flaps are often chosen, which are associated with a high complication rate. Such salvage interventions must be selected so that an amputation is not disadvantageously influenced by the selection of the donor site or is even impossible.

**Methods:**

The three distally based local flaps the sural artery flap, peroneus brevis muscle flap and perforator-based propeller flaps are discussed with respect to placement of the donor site as well as wound healing disorders.

**Results:**

The sural artery flap is disadvantageous as the donor site because the proximal dorsal calf region is affected, which in the case of a below the knee amputation enables soft tissue covering of the stump.

**Conclusion:**

If a local flap is to be used as a salvage surgery in an attempt to prevent a below the knee amputation in a patient who is not suitable for free tissue transfer, special emphasis must be placed on the donor site of this flap. The proximal dorsal aspects of the distal calf are required for covering a potential stump and should not be violated by harvesting a local flap.

## Hinführung zum Thema

Weichteildefekte und Wundheilungsstörungen sind eine große Herausforderung bei der Versorgung offener Frakturen oder deren Folgen. Gerade bei geriatrischen Patienten stellt dies besondere Anforderungen an die rekonstruktive Chirurgie. Auch bei älteren Patienten stellt der mikrovaskuläre Gewebetransfer den Goldstandard dar, ist aber manchmal dennoch nicht möglich [8, 20]. Lokale Lappenplastiken sind unter diesen Voraussetzungen als Alternative mit hohen Komplikationsraten assoziiert und können nicht immer den Erhalt der Extremität ermöglichen. Bei inadäquater Planung und Platzierung der Entnahmestelle kann sogar eine Amputation entscheidend erschwert werden. In diesem Beitrag werden 3 häufig angewendete lokale Lappenplastiken mit Blick auf die besondere Indikationsstellung vorgestellt und deren Anwendung dargelegt.

## Hintergrund

Die Weichgewebsrekonstruktion im Bereich des distalen Unterschenkeldrittels ist eine rekonstruktive Herausforderung, die nur interdisziplinär zu bewältigen ist. Die plastische Chirurgie kooperiert regelhaft mit Fachdisziplinen wie Viszeralchirurgie, Gynäkologie, Dermatologie, Neurochirurgie, Hals-Nasen-Ohren-Heilkunde, aber auch internistischen Disziplinen. Die Zusammenarbeit mit der Unfallchirurgie/Orthopädie ist traditionell sehr eng. In fast keinem anderen Gebiet konnte in Zusammenarbeit eine derartig weitreichende Verbesserung der Versorgungsqualität erzielt werden, die den Erhalt der Extremität ermöglicht [11]. Die aufgrund der veränderten Bevölkerungsstruktur in Zukunft immer komplexer werdende Frakturversorgung älterer Menschen wird ebenso mit einer steigenden Komplexität der Weichgewebsrekonstruktion einhergehen [12, 14]. Daher ist speziell diesem Feld eine hohe Bedeutung in der Zukunft beizumessen. Dass Alter alleine keine Kontraindikation auch für aufwendige mikrochirurgische Weichgeweberekonstruktionen darstellt, ist durch Studien verifiziert [4, 8]. Der freie Gewebetransfer gilt daher als Goldstandard. Die niedrigen Komplikationsraten des freien mikrovaskulären Gewebetransfers auch bei Patienten jenseits des 80. Lebensjahres bedeuten aber nicht, dass der mikrovaskuläre Gewebetransfer immer indiziert und verfügbar ist [8]. Diese Studien zeigen neben der hohen technischen Expertise der Autoren auch die Bedeutung der guten Indikationsstellung in Bezug auf die Patientenselektion, um die Erfolgsquote zu maximieren. Dies bedeutet aber auch, dass primär für den freien Gewebetransfer nichtgeeignete Patienten neben der offenen Wundbehandlung nur noch mit lokalen Lappenplastiken extremitätenerhaltend versorgt werden können. In diesen Fällen kommt die lokale Lappenplastik als „Rettungseingriff“ zum Einsatz, was zwangsläufig mit einer hohen Komplikationsrate einhergeht. Berkara et al. konnten zeigen, dass neben anderen Komorbiditäten das Alter das relative Risiko für den Gewebeverlust beim Einsatz lokaler Lappenplastiken deutlich erhöht [5]. Die teilweise schlechte Reputation lokaler Lappenplastiken ist wohl auch auf die „alternativlose“, teilweise undifferenzierte Indikationsstellung als letzte verfügbare Option zurückzuführen. Grundsätzlich sollten Rettungseingriffe so konzipiert werden, dass weitere Eingriffe dadurch nicht erschwert oder gar unmöglich werden. Daher ist es erforderlich, in die Indikationsstellung zur lokalen Lappenplastik eine im weiteren Verlauf evtl. doch notwendig werdende Amputation einzubeziehen. Somit sollten Verfahren bevorzugt werden, die den proximalen, dorsalen Haut- und Muskelmantel komplett erhalten, um genügend Weichgewebe zur sicheren Stumpfbildung zu erhalten und langfristig die Belastbarkeit des Stumpfes zu gewährleisten. Ziel dieser Studie ist es, gestielte Lappenplastiken zur Rekonstruktion komplexer Weichteildefekte im distalen Unterschenkeldrittel im Hinblick auf ihre Anwendbarkeit zu eruieren, falls der Erhalt der Extremität gefährdet zu sein scheint. Von dieser Situation ist bei allen älteren Patienten auszugehen, die als nichtgeeignet für den freien mikrovaskulären Gewebetransfer angesehen werden, der als Goldstandard gilt.

## Bekannte gestielte Lappenplastiken

Unter vielen gestielten Verfahren haben sich in den letzten Jahrzehnten 3 grundlegend unterschiedliche lokale Lappenplastiken im Bereich des distalen Unterschenkeldrittels klinisch etabliert. Neben der distal gestielten Suralislappenplastik als axiale Lappenplastik konnte sich die gestielte Muskellappenplastik in Form des Peronaeus-brevis-Lappens durchsetzen [1–4, 6, 7]. Als moderne, gestielte Lappenplastik lösten perforatorbasierte Verfahren die unzuverlässigen lokalen fasziokutanen Alternativen ab [11, 19].

### Suralislappenplastik

**Figure 1:**
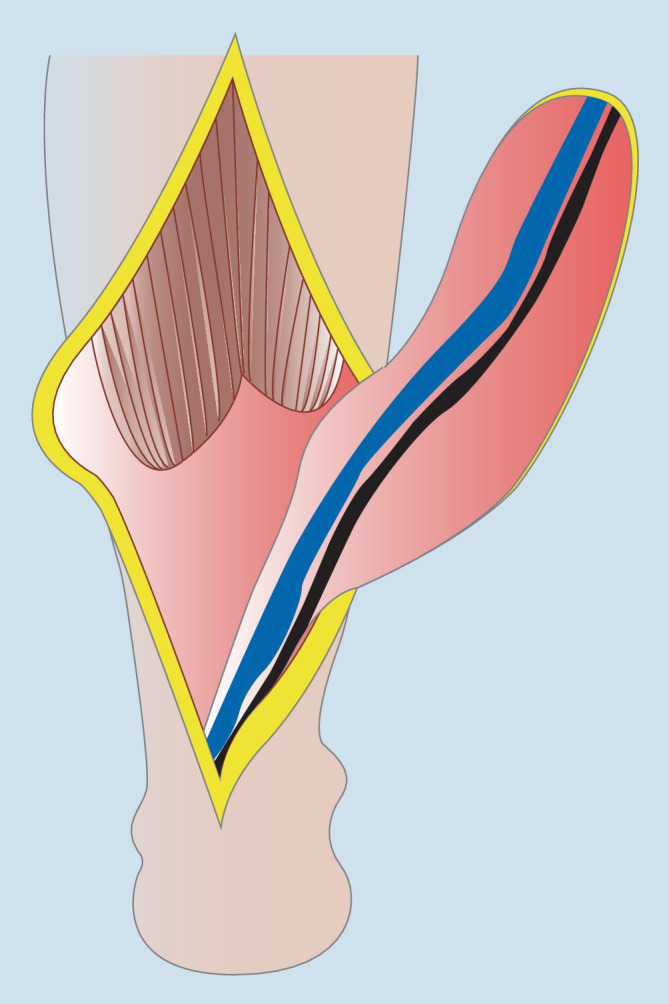

**Figure 2:**
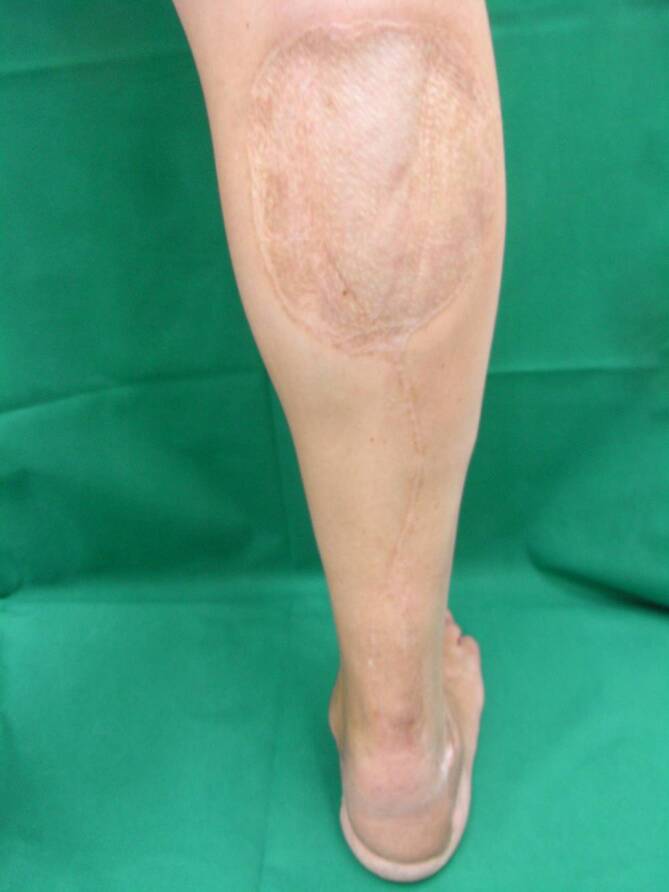

**Figure 3:**
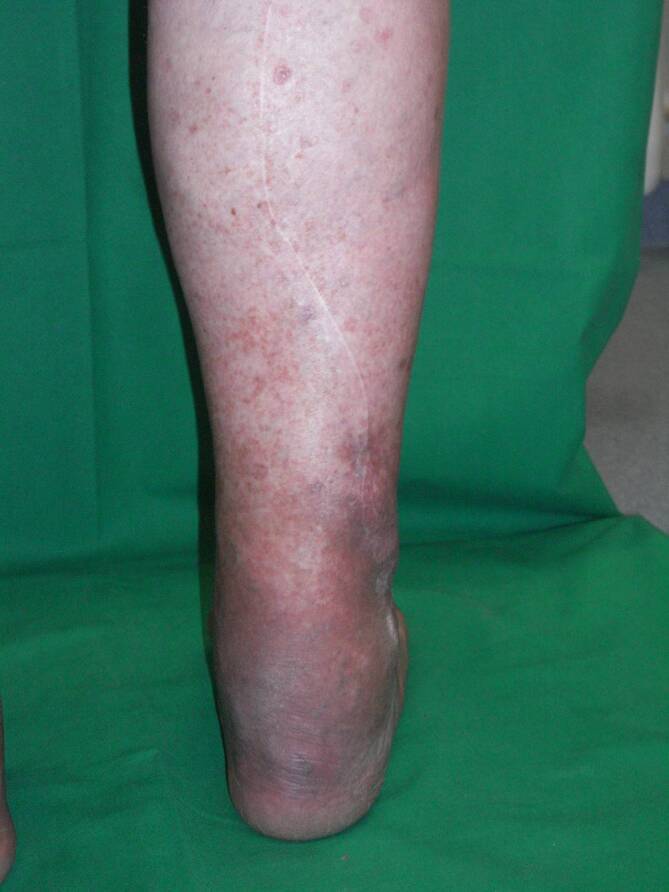

Bei der distal gestielten Suralislappenplastik handelt es sich um eine axiale Lappenplastik, die über die parallel zum N. suralis verlaufende A. suralis retrograd perfundiert wird [1, 2, 18, 20]. Bei dieser schon sehr lange bekannten Lappenplastik ist daher eine Strömungsumkehr erforderlich, um die Perfusion zu sichern. Auch im venösen Abfluss über die V. saphena parva ist eine Strömungsumkehr erforderlich [1, 15, 17]. Die über eine dorsal mittige Inzision gehobene Lappenplastik kann proximal bis in die Kniekehle ausgedehnt werden und somit neben der gesamten Knöchelregion auch kleinere Defekte an der Fußsohle erreichen ([2]; Abb. 1). Neben der klassischen fasziokutanen Variante, die eine Spalthauttransplantation der Entnahmestelle erfordert, ist auch eine adipofasziale Version geläufig [2, 17]. Gerade bei älteren Patienten, bei denen Kontraindikationen für einen freien Gewebetransfer vorliegen, ist die Indikationsstellung für eine Suralislappenplastik als Alternative besonders kritisch zu stellen. Als Kontraindikationen gelten neben der chronisch-venösen Insuffizienz auch die periphere arterielle Verschlusskrankheit (pAVK) und Diabetes mellitus [4, 15]. Diese sind besonders für Wundheilungsstörungen an der Entnahmestelle verantwortlich. Zur Tamponade tieferer Gewebedefekte oder Plombierung einer offenen Gelenkverbindung kann der distal gestielte Suralislappen ebenfalls mit einer Muskelplombe aus dem Gastroknemius gehoben werden [1]. Die allgemeinen Komplikationsraten dieser Lappenplastik variieren stark; bis zu 40 %ige Komplikationsraten sind beschrieben worden [4, 13, 17, 20]. Neben Wundheilungsstörungen im Bereich der Entnahmestelle ist die häufig anzutreffende Minderperfusion der Hautinsel bei der fasziokutanen Version der häufigste Grund für den Lappenverlust. Daher wurde die adipofasziale Variante entwickelt, welche die Komplikationsraten deutlich reduzieren konnte [2, 17]. Dennoch kann auch diese Variante die Reichweite der Lappenplastik nicht erhöhen und somit eine Spitzennekrose verhindern. Da generell bei gestielten Lappenplastiken eine Teilnekrose oder Spitzennekrose häufig funktionell einem kompletten Lappenverlust entspricht, ist diese Lappenplastik sicher nur bei Defekten bis in die Knöchelregion anwendbar. Den großen Nachteil dieser Lappenplastik in der Konstellation einer im weiteren Verlauf evtl. erforderlichen Amputation stellt die Entnahmestelle dar. Die fasziokutane Version der Suralislappenplastik platziert die mit Spalthaut gedeckte Entnahmestelle im dorsalen proximalen Bereich der Wade (Abb. 2). Die Entnahmestelle kommt somit im Fall einer Unterschenkelamputation direkt über dem knöchernen Stumpf am Apex der Hauptbelastungszone zu liegen. Die adipofasziale Variante erfordert zwar keine Hauttransplantation, kompromittiert allerdings den dorsalen Weichgewebsmantel durch die nun mittig in identischer Position liegende Narbe (Abb. 3). Aufgrund der Entnahme der tiefen, adipofaszialen Gewebeschicht wird auch die Hautperfusion durch die Durchtrennung kleiner muskulokutaner Perforatoren potenziell eingeschränkt. Bei den durch die Prothesenversorgung zu erwartenden hohen Druckbelastungen stellt die mittig liegende Narbe somit einen Prädilektionsort für Druckulzerationen dar. Somit ist von dieser Lappenplastik als letzter Versuch der Weichgewebsrekonstruktion vor einer drohenden Amputation abzuraten.

### Peronaeus-brevis-Muskellappenplastik

**Figure 4:**
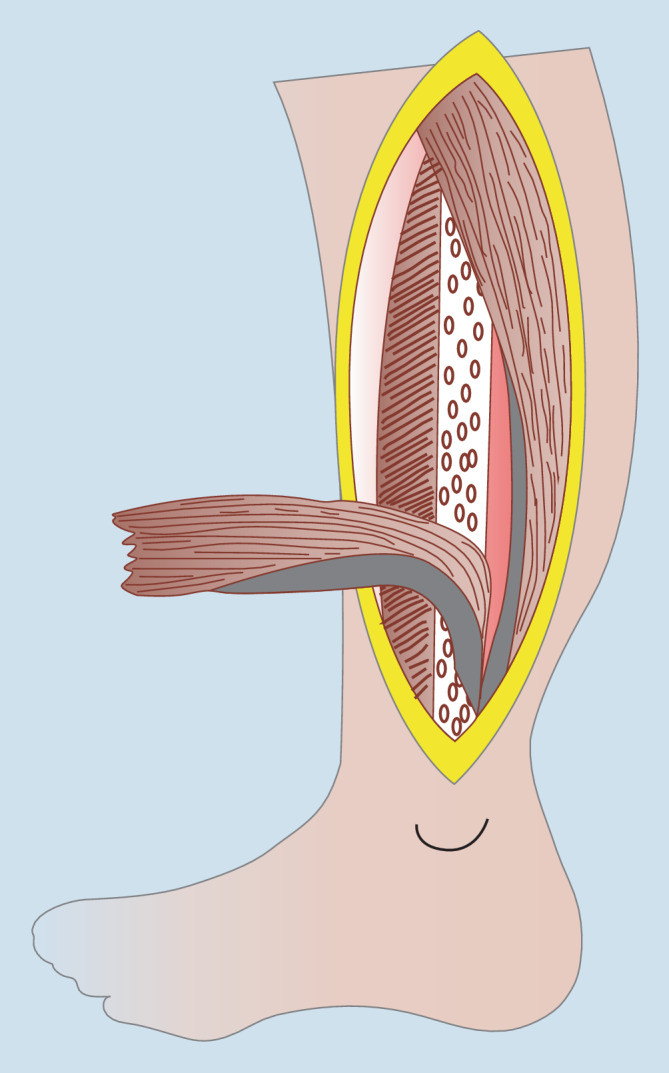

**Figure 5:**
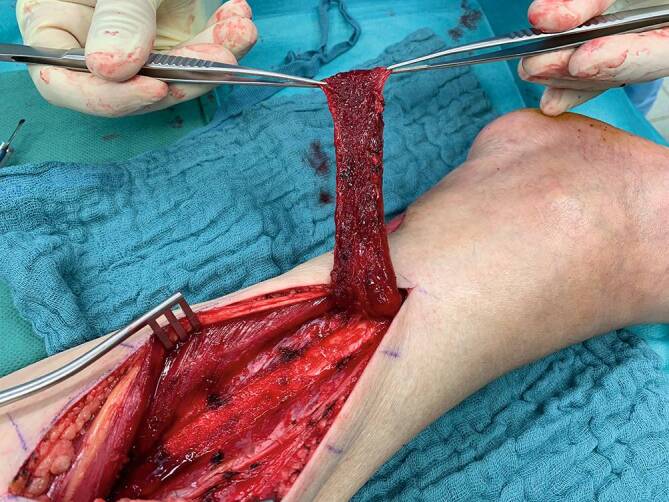

Lokal gestielte Muskellappenplastiken sind rar in der Knöchelregion. Die Peronaeus-brevis-Muskellappenplastik stellt die beste Option in dieser Region in Bezug auf die Perfusionssicherheit, Reichweite und den Funktionsverlust an der Entnahmestelle dar ([3, 6, 7]; Abb. 4). Mit einer maximalen Länge von 20 cm können Defekte im Bereich des Malleolus lateralis, der ventralen Tibia und dorsal im Bereich der Achillessehne adressiert werden [6]. Besonders längsorientierte, schmale Defekte bis 40 cm^2^ Größe sind geeignet; größere Defekte im Bereich des Malleolus medialis können mit dieser Lappenplastik nur inadäquat erreicht werden. In Bezug auf die Perfusion ähnelt die M.-peronaeus-brevis-Lappenplastik dahingehend der Suralislappenplastik, dass auch hier ist eine Strömungsumkehr notwendig ist. Da der M. peronaeus brevis sowohl von Perforatoren aus der A. tibialis anterior als auch der A. fibularis versorgt wird, ist die Muskellappenplastik auch beim Verschluss eines dieser Gefäße anwendbar. Diese werden durch die Platzierung des Umschlagpunktes 6 cm proximal der Fibulaspitze sicher geschont [3, 13]. Da es sich prinzipiell um einen kleinen Muskel handelt, der nur kleinere Defekte gut decken kann, wurde das „open book splitting“ propagiert, um breitere Defekte sicher verschließen zu können. Dieses Verfahren ist aber gerade bei älteren Patienten zurückhaltend anzuwenden, da neben der zentralen Ausdünnung der Lappenplastik auch Teilnekrosen bei Verschluss von einem der Hauptgefäße möglich sind. Neben dem minimalen Funktionsverlust und der Möglichkeit der Totraumtamponade durch die Muskulatur ermöglicht die laterale Platzierung des Zugangsweges an der Entnahmestelle den Einsatz dieser Lappenplastik als Rettungseingriff bei Patienten vor einer drohenden Unterschenkelamputation, da der gesamte dorsale proximale Weichteilmantel des Unterschenkels geschont werden kann (Abb. 5). Bei kleineren Defekten im Bereich des Außenknöchels und der ventral und dorsal angrenzenden Region stellt diese Lappenplastik eine favorisierte Option dar [13].

### Mediale, perforatorbasierte Propellerlappenplastik

**Figure 6:**
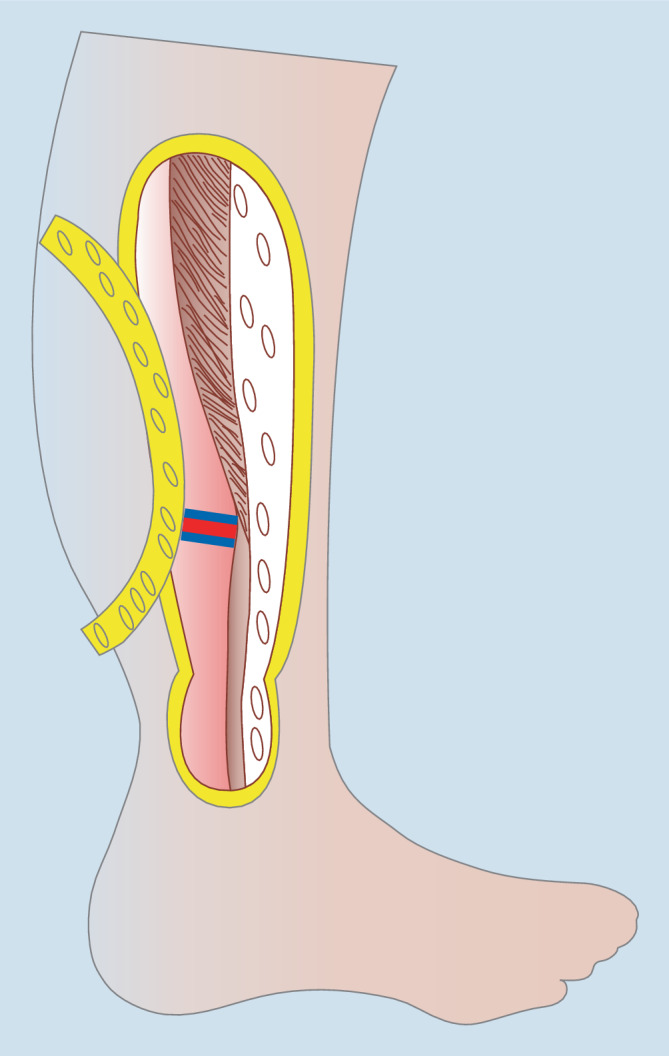

**Figure 7:**
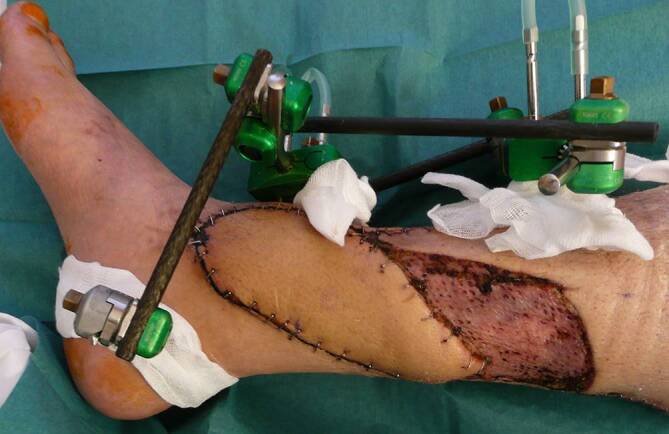

Propellerlappenplastiken als eigene Untergruppe der perforatorbasierten lokalen Lappenplastiken sind seit etwa 2 Dekaden klinisch etabliert und haben die früher populären „Random-pattern“-Lappen weitestgehend abgelöst [19]. Da eine sichere Perfusion einer großen Lappenplastik über einen einzelnen Perforator möglich ist, ermöglicht sich eine sichere Lappendeckung überall dort, wo ein solcher Perforator angetroffen werden kann ([11, 16]; Abb. 6). Propellerlappenplastiken sind besonders für mediale Defekte gut geeignet, da größere Perforatoren der A. tibialis posterior regelhaft 5 cm, 10 cm und 15 cm proximal der Spitze des Malleolus medialis vorhanden sind und mittels Dopplersonographie gefunden werden können [10, 16]. Die präoperative Diagnostik mittels farbkodierter Duplexsonographie (FKDS) erlaubt eine verlässlichere Darstellung der Perforatoren als der Einsatz des Handdopplers, der häufig zu falsch-positiven Befunden führt [10]. Auch wenn die Hauptgefäße in diesem Bereich häufig arteriosklerotisch geschädigt sind, können die Perforatoren verschont sein, was die Torsion des Gefäßstiels erlaubt. Die konische Form des Unterschenkels bedingt auch nach distal immer kürzer werdende Perforatoren, sodass einfache Vorschublappen kaum möglich sind. Anders als bei der Suralis- oder Peronaeus-brevis-Lappenplastik bleiben die arterielle und venöse Flussrichtung erhalten. Da allerdings die venöse Drainage der Hautinsel hauptsächlich über die subkutanen Venen erfolgt, kann eine Insuffizienz des tiefen Systems zu venöser Stase und evtl. Lappennekrose führen. Da Perforatoren der A. tibialis anterior und auch der A. fibularis kaliberschwächer und deutlich variabler sind, wird die Propellerlappenplastik hauptsächlich medial, basierend auf Perforatoren der A. tibialis posterior, angewendet [9, 11]. Neben Defekten im Bereich des Malleolus medialis können auch dorsal und ventral gelegene Defekte gut erreicht werden. Da die Entnahmestelle immer longitudinal zur Beinachse platziert werden sollte, ist bei dorsalen und ventralen Defekten ein Rotationsradius von weniger als 180° erforderlich, was die Perfusion einer Propellerlappenplastik verbessert [16, 21]. Anders als bei der Peronaeus-brevis-Muskellappenplastik kann die Lappenplastik auch breite Defekte gut verschließen, was allerdings den Einsatz eines Spalthauttransplantats an der Entnahmestelle erfordert. Daher ist speziell als Rettungseingriff der Einsatz der Propellerlappenplastik bei Defekten über 6 cm Breite umsichtig zu planen und die Entnahmestelle eher ventral zu platzieren, um den dorsalen Anteil des proximalen Unterschenkels zu schonen. Die mediale Propellerlappenplastik stellt für mediale Defekte und auch ventrale und dorsale Defekt auf Knöchelhöhe eine gute lokale Alternative dar, die die gesamten dorsalen Anteile des proximalen Unterschenkels schont und erhält (Abb. 7).

## Ausblick

Aufgrund der demografischen Entwicklung nimmt die Bedeutung der Frakturversorgung geriatrischer Patienten zu. Gerade die Häufung der für die Wundheilung relevanten Nebenerkrankungen bedingt Weichgewebsdefekte und Wundheilungsstörungen, die einer plastisch-chirurgischen Rekonstruktion bedürfen. Nicht immer ist der freie, mikrovaskuläre Gewebetransfer durchführbar, sodass manchmal auf lokale Lappenplastiken zurückgegriffen wird. Grundsätzlich ist dem Patienten und den Angehörigen klar zu kommunizieren, dass es sich in solchen Ausgangslagen immer um Situationen handelt, in welcher der Erhalt der Extremität nicht sicher zu gewährleisten ist. Da hierbei mit einer erhöhten Komplikationsrate gerechnet werden muss, ist die Planung dieser Lappenplastiken ebenfalls auf die Möglichkeit einer späteren Amputation auszurichten, um nicht durch die Entnahmestelle die sichere Weichteildeckung des Stumpfes zu gefährden oder gar eine deutlich proximal gelegenere Amputationshöhe erforderlich zu machen. Vor diesem Hintergrund ermöglicht der Einsatz lokaler Lappenplastiken den langfristigen Erhalt der unteren Extremität auch beim alten Menschen. Die Amputation stellt ebenfalls eine Therapiemöglichkeit dar und ist nicht als Therapieversagen anzusehen. Dennoch sollte aufgrund der bekannten Probleme bei der Prothesenversorgung älterer Patienten der Erhalt der Extremität angestrebt werden.

## Fazit für die Praxis

Der freie mikrovaskuläre Gewebetransfer ist auch bei alten Menschen im Bereich des distalen Unterschenkels ein sicheres Verfahren.

Bei Kontraindikationen können lokale Lappenplastiken wie die Peronaeus-brevis-Muskellappenplastik für lateral und kleine ventrale und dorsale Defekt zum Einsatz kommen. Bei medialen und größeren ventralen und dorsalen Defekten stellt die perforatorbasierte Propellerlappenplastik eine gute Möglichkeit dar.

Da die Entnahmestelle der Suralislappenplastik im Bereich des proximalen, dorsalen Unterschenkels das für eine potenzielle Stumpfbildung wichtige Areal kompromittiert, ist die Indikation kritisch zu stellen.
